# P-294. Utilization and Barriers to Pharmacist-Prescribed HIV Post-Exposure Prophylaxis (PEP) in New Mexico

**DOI:** 10.1093/ofid/ofaf695.515

**Published:** 2026-01-11

**Authors:** Carly Cloud Floyd, Bernadette Jakeman

**Affiliations:** University of New Mexico College of Pharmacy | South Central AIDS Education & Training Center, Albuquerque, NM; University of New Mexico, Albuquerque, NewMexico

## Abstract

**Background:**

New HIV infection rates in the United States (US) have plateaued in recent years. According to the latest US CDC report, there were approximately 38,000 new HIV diagnoses in 2022. To combat the spread of HIV, the US Department of Health and Human Services introduced the Ending the HIV Epidemic (EHE) initiative, aiming to reduce new infections to 3,000 by 2030. In line with national EHE efforts, in 2020 the state of New Mexico (NM) authorized pharmacists to interpret HIV screenings and prescribe HIV post-exposure prophylaxis (PEP) under regulation 16.19.26.14. The objectives of this study were to: 1) Describe utilization of HIV testing and PEP prescribing by NM pharmacists; and 2) Identify barriers with service implementation.

**Methods:**

From May to August 2024, study investigators conducted an anonymous online REDCap survey of NM pharmacists who completed the pharmacist PEP training from 10/01/2021 to 10/31/2023. Data collected included: demographics, reasons for obtaining certification, assessment of the training program quality, self-perceived competency after training, recommendations for improving the program, current or historical practices, reasons for not utilizing the certification, and noted barriers to implementation and utilization.

**Results:**

Of 122 trained pharmacists, 17 (13.9%) participated in the survey. The majority of respondents were female and from the Albuquerque metro area. Participants most frequently cited skill development and a desire to improve public health as motivations for certification. However, only two pharmacists reported they were currently offering HIV PEP services. Importantly, neither had performed HIV testing or prescribed HIV PEP at the time of survey completion. Key barriers to providing HIV PEP services included employer lack of support (29%), staffing issues (29%), and reimbursement issues (24%).

**Conclusion:**

As readily accessible healthcare professionals, pharmacists have significant potential to contribute to the goals of the EHE initiative. However, the survey findings reveal substantial barriers to pharmacist-prescribed PEP, including reimbursement issues and lack of employer support. To enable a more coordinated delivery of HIV PEP in community pharmacies, policy changes at state and national levels are essential.

**Disclosures:**

Bernadette Jakeman, PharmD, CEImpact: Honoraria|Clinical Care Options, LLC: Honoraria|Merck: Grant/Research Support|PTCE: Honoraria|Wolters-Kluwer, Lexidrug: Advisor/Consultant

